# Stereotactic robotic body radiotherapy for patients with oligorecurrent pulmonary metastases

**DOI:** 10.1186/s12885-020-06906-1

**Published:** 2020-05-08

**Authors:** Patrick Berkovic, Akos Gulyban, Gilles Defraene, Laurie Swenen, David Dechambre, Paul Viet Nguyen, Nicolas Jansen, Carole Mievis, Pierre Lovinfosse, Levente Janvary, Maarten Lambrecht, Gert De Meerleer

**Affiliations:** 1 Department of Radiation Oncology, University Hospitals Leuven, Herestraat 49, 3000 Leuven, Belgium; 2 Department of Radiation Oncology, University Hospital of Liège, Avenue de L’Hòpital 1, 4000 Liège, Belgium; 3 Medical Physics Department, Jules Bordet Institute, Université Libre de Bruxelles, 1 rue Héger-Bordet, 1000 Brussels, Belgium

**Keywords:** Lung-Oligometastases-Oligorecurrence-Cyberknife-prognostic factors-outcome

## Abstract

**Background:**

Our aim is to report treatment efficacy and toxicity of patients treated by robotic (Cyberknife®) stereotactic body radiotherapy (SBRT) for oligorecurrent lung metastases (ORLM). Additionally we wanted to evaluate influence of tumor, patient and treatment related parameters on local control (LC), lung and distant progression free- (lung PFS/Di-PFS) and overall survival (OS).

**Methods:**

Consecutive patients with up to 5 ORLM (confirmed by FDG PET/CT) were included in this study. Intended dose was 60Gy in 3 fractions (prescribed to the 80% isodose volume). Patients were followed at regular intervals and tumor control and toxicity was prospectively scored. Tumor, patient and treatment data were analysed using competing risk- and Cox regression.

**Results:**

Between May 2010 and March 2016, 104 patients with 132 lesions were irradiated from primary lung carcinoma (47%), gastro-intestinal (34%) and mixed primary histologies (19%). The mean tumor volume was 7.9 cc. After a median follow up of 22 months, the 1, 2 and 3 year LC rate (per lesion) was 89.3, 80.0 and 77.8% respectively. The corresponding (per patient) 1, 2 and 3 years lung PFS were 66.3, 50.0, 42.6%, Di-PFS were 80.5, 64.4, 60.6% and OS rates were 92.2, 80.9 and 72.0% respectively. On univariable analysis, gastro-intestinal (GI) as primary tumor site showed a significant superior local control versus the other primary tumor sites. For OS, significant variables were primary histology and primary tumor site with a superior OS for patients with metastases of primary GI origin. LC was significantly affected by the tumor volume, physical and biologically effective dose coverage. Significant variables in multivariable analysis were BED prescription dose for LC and GI as primary site for OS. The vast majority of patients developed no toxicity or grade 1 acute and late toxicity. Acute and late grade 3 radiation pneumonitis (RP) was observed in 1 and 2 patients respectively. One patient with a centrally located lesion developed grade 4 RP and died due to possible RT-induced pulmonary hemorrhage.

**Conclusions:**

SBRT is a highly effective local therapy for oligorecurrent lung metastases and could achieve long term survival in patients with favourable prognostic features.

## Background

Pulmonary tissue is a frequent site of metastatic seeding, with epithelial malignancies and sarcoma having a high tendency to develop lung metastases [1, 2]. Resection of lung metastases (metastasectomy) has become a widespread and accepted standard therapy [2, 3]. However, evidence for pulmonary metastasectomy is weak as no randomized clinical trials exist to support its benefit. In case of oligometastatic lung disease (OMLD) [4], less invasive interventions such as radiofrequency- and microwave ablation and stereotactic body radiotherapy (SBRT) have been described as a valid alternative to surgery [5].

SBRT for OMLD yields high local control and low toxicity rates; in case of limited (maximal 5) lung metastases, the 2-year local control varies between 78 and 96% [6–10]. The term OMLD has been further refined with the concept of oligorecurrent lung metastases (ORLM), defined as the development of metachronous lung oligometastases after radical treatment of the primary tumor [11]. This refinement rules out patients presenting with OMLD at initial diagnosis or OMLD with an uncontrolled primary tumor. Several studies suggest an improved outcome for patients with ORLM when compared to synchronous OMLD [12, 13].

The major challenge when delivering SBRT to lung metastases is to manage respiratory motion of both tumor and normal tissues. Breathing control strategies such as breath-hold, gating, tumor tracking and motion management techniques have been implemented to deal with this challenge [14, 15]. One of those strategies is the Cyberknife® synchrony (Accuray Inc., Sunnyvale, CA), which performs real-time tumor tracking. This allows for a high level of precision while treating the patient in the comfort of free breathing [16].

The primary aim of this retrospective study is to report on local control, lung and distant progression free survival and overall survival of patients with ORLM treated with SBRT using the Cyberknife®. The secondary aim is to report on SBRT-induced acute and late toxicity and to identify prognostic factors influencing the treatment outcome.

## Methods

### Patient, tumor and treatment characteristics

Consecutive patients with up to 5 ORLM were included in this study for Cyberknife treatment at the Liege University Hospital, Belgium. All patients were referred for SBRT after full staging including baseline registration of the pulmonary function, chest and abdominal diagnostic computed tomography (CT) and [18F]-fluorodeoxyglucose (FDG) positron emission tomography (PET)-CT imaging confirming local control at the primary tumor site and the absence of non-pulmonary metastases. Indications for SBRT were discussed and approved by the multi-disciplinary tumor board. Patients were informed of the intent, possible side effects and practical modalities and provided consent for treatment prior to SBRT. Since the study was retrospective, informed consent was waived. We included patients with both de-novo metachronous (57 lesions)-and repeat ORLM (75 lesions) according to the definition proposed by Guckenberger et al. [17]. Lesions in any location within the lung were included in this analysis, irrespective of prior treatments including systemic therapy, surgery or previous radiotherapy for their primary lesion or prior metastases (Table 1). Exclusion criteria were heavily compromised pulmonary function tests with grade IV COPD, co-existing ILD and IPF, pleural effusion, metastases with diameter > 6 cm and patients with a life expectancy of less than 6 months.

**Table 1:** **Patient and tumor characteristics**

	Per patients (1st lesion)	Per lesion/treatment
**Gender**		
Female	49 (47.1%)	61 (46.2%)
Male	55 (52.9%)	71 (53.8%)
**Age (at SBRT, years)**		
Median (range)	66.4 (28.2–87.6)	65.2 (28.2–86.6)
**Location of the lesion**		
Periferal		80 (61%)
Central		52 (39%)
**Primary sites**		
Lung	49 (47.1%)	
Gastro-intestinal	35 (33.7%)	
Other	20 (19.2%)	
**Primary histology**		
Adenocarcinoma	67 (64.4%)	86 (65.2%)
Other	37 (35.6%)	46 (34.8%)
**Lesion treated at same time (on same CT)**		
1	82 (78.8%)	
2	16 (15.4%)	
3	4 (3.8%)	
4	2 (1.9%)	
**Prior chemotherapy for primary tumor**		
Yes	25 (24%)	27 (20.5%)
No	79 (76%)	105 (79.5%)
**Performance status**		
0	24 (23.1%)	29 (22%)
1	71 (68.3%)	91 (68.9%)
2	9 (8.7%)	12 (9.1%)
**Previous treatments (per patient)**^**a**^		
Surgery	45 (34.1%)	
Chemotherapy	47 (35.6%)	
Radiotherapy	42 (31.8%)	
Other	57 (43.2%)	

^***a***^***combined treatments are counted separately***

### Cyberknife planning and treatment

A majority of patients had radio-opaque 3 mm long gold fiducials (Goldlock, Beampoint, Kista, Sweden) inserted alongside the tumor by CT-guided transthoracic punction by a dedicated interventional radiologist and placed according to Accuray’s guidelines [18]. Treatment preparation, planning characteristics and tracking options of the Cyberknife system have been previously described [19, 20]. In short, real time tracking was preferred either on implanted fiducials (Synchrony®) or - in case of contra-indications and clear identification of tumor projection on both orthogonal detector panels –using image guidance (Xsight Lung®), leaving the vertebra based position verification (XsightSpine®) as last option. Left and right lung, oesophagus, heart, thoracic wall or ribs, trachea, spinal canal, great vessels, ipsilateral brachial plexus (for sulcus superior lesions) and a 4 mm thick skin area were delineated as organs at risk. The expiration CT scan was used as reference for treatment planning purpose. The gross tumor volume (GTV) was defined on this scan using mutual information from additional image modalities, and was expanded with 3 mm margin to create a CTV followed by manual modification in presence of adjacent anatomical borders. The planning target volume (PTV) was created by adding a 2 mm uniform margin around the CTV. Intended prescription was 60 Gy in 20 Gy per fraction for the 80% isodose, with at least 95% PTV coverage. The organs at risk (OAR) dose constraints reported by Timmerman [21] were always respected, using a risk-adapted fractionation scheme concept when necessary. The estimated fraction duration was kept below 70 min and the treatment sessions were delivered at an interval of minimum 40 h. Details on the dose calculation algorithms (Ray Tracing followed by recalculation with Monte Carlo) can be found in our previous work [20, 22].

### Follow up and toxicity evaluation

Patients were evaluated for acute toxicity after the last fraction, at 2 weeks, and then in function of the referring team at an interval of 2 to 4 months. Treatment response was evaluated at this interval by serial contrast enhanced spiral CT and was defined according to the Response Evaluation Criteria in Solid Tumors version 1.1 [23]. In case of (serial) CT-features suggesting progression of the treated lesion, a FDG PET/CT was performed followed by a fine needle biopsy (if feasible) to confirm local failure.

Local control (LC) was defined as one of the following: complete response, partial response or stable disease (CR, PR or SD). Recurrences within the PTV were classified as local failure whereas recurrences in the same or other lobe, beyond the PTV were considered as metachronous pulmonary metastases. Extra-pulmonary recurrences were classified as distant metastases. Toxicity was evaluated using the Common Terminology Criteria for Adverse Events v4.0 [24]. Toxicities occurring less than or equal to 3 months following SBRT were considered as acute whereas toxicities arising after 3 months were considered as late.

### Data analysis

LC was analysed in a competing risk regression analysis. Lung and distant progression free-survival (L-PFS, Di-PFS) and overall survival (OS) curves were evaluated by the (competing risk and) Kaplan-Meier analysis method. Data for LC were determined for each lesion separately, while the L-PFS, Di-PFS and OS were determined per patient originated from the day of the first SBRT treatment. Univariable analysis was performed using Gray’s test and the log rank test for competing risk and Kaplan-Meier analysis, respectively. Clinical parameters were analysed for all outcome parameters. For LC, additional investigation included treatment planning related parameters such as number of fractions, target volumes, physical and biological effective dose (BED10 considering α/β = 10) and coverages for GTV, CTV and PTV in a systematic manner. Finally, multivariable analyses started with a preselection of the parameters based on their univariable *p* value (*p* < 0.10) and their correlation (Pearson’s correlation r < 0.7). The Fine and Gray and the Cox regression methods were followed for competing risk and Kaplan-Meier analysis, respectively. For all tests a *p*-value < 0.05 was considered as statistically significant using Python packages (pandas 0.21.0, scipy 0.18.0 and lifelines 1.9.4.0) and R software (v.3.4.4, R Foundation for Statistical Computing, Vienna, Austria).

## Results

Between May 2010 and March 2016 a total of 104 patients with 132 lesions were irradiated in 106 treatments. Sixteen, four and two patients were treated for respectively two, three and four synchronous lesions. In case of a second de-novo metachronous-or repeat ORLM, a new SBRT session was the treatment of choice in 42 patients. Table 1 contains detailed information on patient and tumor characteristics.

### Treatment characteristics

The mean GTV and PTV volumes were 7.9 cc (90% CI, 0.4–36.1) and 25.3 cc (90% CI, 3.9–74.7) respectively. All treatments were delivered 3x/week on every other day in three or five fractions for respectively 59 and 41% of the patients to an average PTV median dose (D50) of 62.4 Gy (90% CI, 44.4–69.4). This corresponds to an average PTV_BED10_ of 177.6 Gy (90% CI, 83.9–229.7). The mean GTV and PTV D98% were 58.3 Gy (90% CI, 36.2–70.1) and 52.5 Gy (90% CI, 31.6–61.7) respectively. Each treatment was delivered by an average of 120 (35–230) beams during a median of 45 min (30–63 min) session. Further dosimetric parameters are presented in Table 2.

**Table 2:** Tumor and treatment related parameters

	Per patients (1st lesion)	Per lesion (treatment)
**Prescription**		
3 × 6.67Gy (BED10: 33.36 Gy)	0 (0%)	2 (1.5%)
5x7Gy (BED10: 59.5 Gy)	1 (1%)	3 (2.3%)
5x8Gy (BED10: 72 Gy)	5 (4.8%)	5 (3.8%)
5x9Gy (BED10: 85.5 Gy)	5 (4.8%)	7 (5.3%)
5x10Gy (BED10: 100 Gy)	13 (12.5%)	15 (11.4%)
5x11Gy (BED10: 115.5 Gy)	9 (8.7%)	11 (8.3%)
5x12Gy (BED10: 132 Gy)	10 (9.6%)	11 (8.3%)
3x15Gy (BED10: 112.5 Gy)	2 (1.9%)	4 (3.0%)
3x17Gy (BED10: 137.7 Gy)	3 (2.9%)	1 (0.8%)
3x20Gy (BED10: 180 Gy)	56 (53.8%)	73 (55.3%)
**Tracking**		
Synchrony	44 (42.3%)	56 (42.4%)
Xsight Lung	13 (12.5%)	16 (12.1%)
Xsight Spine	47 (45.2%)	60 (45.5%)
**RECIST**		
Complete Remission	63 (60.6%)	76 (57.6%)
Partian Remission	12 (11.5%)	15 (11.4%)
Stable Disease	4 (3.8%)	5 (3.8%)
Progressive Disease	25 (24%)	36 (27.3%)
**Treatment execution**		
		**Per lesion/treatment**
**Nr. of beams**		
Average (Range)		120 (35–230)
**Total MU**		
Average (Range)		37,821 (11902–89,666)
**Volumes (Mean, (Range) in cm3)**		
GTV		7.9 (0.3–105.2)
CTV		19.0 (1.4–147)
PTV		25.3 (2.8–178.7)
**Dosimetric parameters (Mean, (95% CI) in Gy)**		
GTV-D98		58.3 (30.0–70.2)
GTV-D90		60.7 (33.3–71.6)
GTV-Dmean		64.7 (43.2–73.2)
GTV-D2		68.9 (48.5–75.2)
GTV-BED10Gy D98		162.6 (49.1–234.6)
GTV-BED10Gy D95		167.4 (52.8–238.3)
GTV-BED10Gy D90		172.4 (55.7–242.6)
GTV-BED mean		189.8 (80.8–251.8)
GTV-BED10Gy D2		208.8 (95.5–264.0)
CTV-D98		55.6 (27.5–66.4)
CTV-D90		59.0 (35.0–68.4)
CTV-Dmean		63.6 (43.3–71.4)
CTV-BED10Gy D98		150.8 (44.0–213.6)
CTV-BED10Gy D95		157.5 (51.3–218.4)
CTV-BED10Gy D90		163.9 (61.3–224.2)
CTV-BED mean		184.4 (81.0–241.4)
PTV-D98		52.5 (23.7–61.8)
PTV-D90		56.3 (32.1–64.5)
PTV-Dmean		62.0 (42.6–69.5)
PTV-BED10Gy D98		137.6 (37.0–189.2)
PTV-BED10Gy D95		144.7 (42.4–197.4)
PTV-BED10Gy D90		151.7 (55.0–203.4)
PTV-BED mean		176.5 (79.0–231)

### Treatment efficacy

**Fig. 1:**
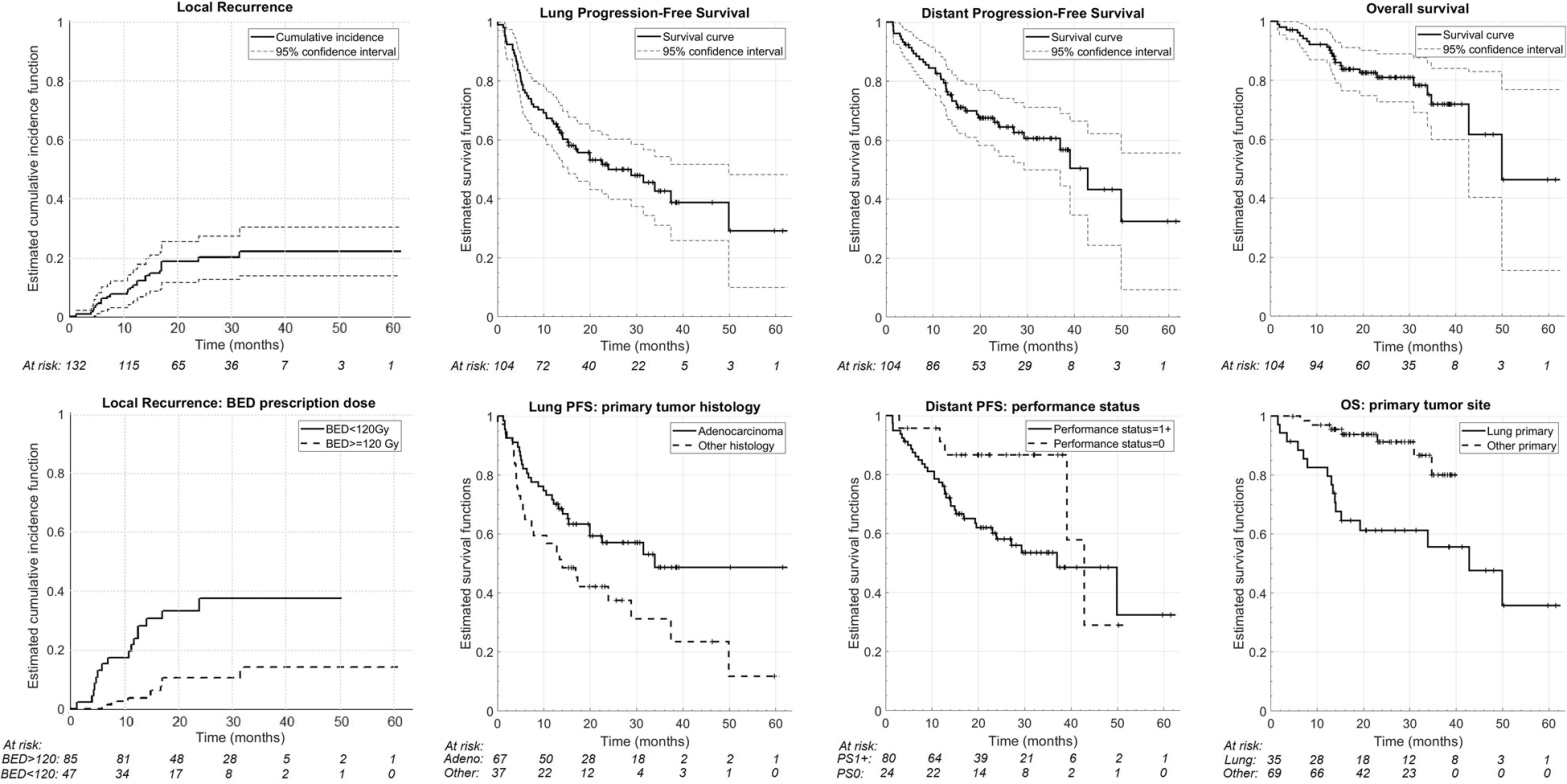
Cumulative incidence of local recurrence (competing risk analysis) and Kaplan-Meier curves for lung and distant progression free survival and overall survival. In the upper row curves describing the whole cohort. In the lower row, subcohorts based on variables significant in multivariable analysis

After a median follow up of 22.0 months (range: 1.5–61.0), the number of patients (lesions) at risk were 83 (104), 43 (25) and 16 (17) at 1, 2 and 3-year respectively. LC rate (based on the RECIST criteria, Table 2) - following each lesion separately - was 89.3% at first, 80.0% at second and 77.8% at third year. The corresponding – per patient – lung PFS and the Di-PFS rates at 1, 2 and 3 years were 66.3, 50.0, 42.6 and 80.5, 64.4, 60.6% respectively. Associated OS rates at 1, 2 and 3 years were 92.2, 80.9 and 72.0% (Fig. 1). Associations of variables with the outcome parameters are summarized in Tables 3 and 4. In univariable analysis for LC, all clinical variables were marginally significant while gastro-intestinal (GI) as primary tumor site showed a significant superior local control versus the other primary tumor sites (*p* = 0.029). For lung PFS, a primary histology of adenocarcinoma (*p* = 0.023) and a primary tumor site other than lung (*p* = 0.043) were significantly superior. Regarding distant PFS, only PS (*p* = 0.060) was marginally significant in univariable analysis (no multivariable analysis required). For OS, significant variables were primary histology (*p* = 0.009) and primary tumor site with a superior OS for patients with metastases of primary GI origin (*p* = 0.0015). GTV volumes correlated with local control (*p* = 0.0021). For GTV, CTV and PTV a physical dose coverage of 60Gy to at least 95% of the volume showed significant effect on the LC. A biologically effective dose coverage (α/β = 10) of 160GyBED10 for both GTV and CTV to at least 95% of the volume was a statistically significant threshold for LC. GTV’s near maximum physical (D2) and biologically effective (D2_BED10_) doses of 65Gy and 165Gy_BED10_ respectively significantly impacted LC. Variables which remained significant in multivariable analysis (Table 5) were BED prescription dose (> 120 Gy vs < 120 Gy) for superior LC (*p* = 0.025) and GI as primary site for superior OS (*p* = 0.05).

**Table 3:** Univariable associations of outcome measures and clinical variables: Gray’s test for LC, logrank test for lung PFS, distant PFS and OS

*Endpoint*	Local control		Lung PFS		Distant PFS		Overall survival	
*Clinical variables*	Hazard ratio (95% CI)	*p* value	Hazard ratio (95% CI)	*p* value	Hazard ratio (95% CI)	*p* value	Hazard ratio (95% CI)	*p* value
Primary histology (adeno vs other)	0.52 (0.24–1.12)	0.093	0.49 (0.27–0.87)	0.023	0.73 (0.38–1.41)	0.44	0.28 (0.12–0.67)	0.009
Primary tumor site (lung vs other)	1.94 (0.90–4.16)	0.10	1.88 (1.06–3.33)	0.043	1.66 (0.84–3.29)	0.20	5.77 (2.39–13.94)	< 0.001
Primary tumor site (GI vs other)	0.36 (0.15–0.90)	0.029	0.66 (0.39–1.14)	0.17	0.86 (0.45–1.64)	0.77	0.24 (0.10–0.54)	0.0015
Prior chemotherapy (yes vs no)	3.25 (0.79–13.50)	0.084	1.09 (0.59–2.02)	0.90	0.96 (0.46–1.98)	0.95	0.78 (0.31–2.01)	0.79
Age > 65 (yes vs no)	0.49 (0.21–1.14)	0.090	0.94 (0.55–1.60)	0.92	0.65 (0.34–1.24)	0.25	0.95 (0.42–2.16)	0.93
Performance status (1+ vs 0)	3.63 (0.88–14.90)	0.056	0.76 (0.40–1.46)	0.52	2.10 (1.04–4.25)	0.060	2.58 (1.01–6.58)	0.082
Gender (male vs female)	2.26 (0.94–5.44)	0.060	0.93 (0.54–1.59)	0.90	0.92 (0.49–1.72)	0.92	0.85 (0.37–1.93)	0.86

**Table 4:** Univariable associations (Gray’s test) of LC and dosimetric/treatment variables

*Endpoint*	Local control	
*Dosimetric variables*	Hazard ratio (95% CI)	*p* value
Number of fractions (> 3 vs 3)	1.93 (0.89–4.19)	0.097
BED prescription (> 120 Gy vs < 120 Gy)	0.25 (0.11–0.56)	< 0.001
Tracking (Synchrony vs XSight L./Sp.)	1.39 (0.60–3.24)	0.45
GTV volume (> 10 cc vs < 10 cc)	3.46 (1.55–7.69)	0.0021
CTV volume (> 20 cc vs < 20 cc)	2.14 (0.97–4.75)	0.063
PTV volume (> 25 cc vs < 25 cc)	2.00 (0.91–4.39)	0.085
GTV mean dose (> 70 Gy vs < 70 Gy)	0.22 (0.079–0.63)	0.0025
CTV mean dose (> 65 Gy vs < 65 Gy)	0.33 (0.15–0.73)	0.0052
PTV mean dose (> 65 Gy vs < 65 Gy)	0.33 (0.15–0.73)	0.0052
GTV D95 (> 60 Gy vs < 60 Gy)	0.36 (0.16–0.79)	0.0096
CTV D95 (> 60 Gy vs < 60 Gy)	0.40 (0.18–0.88)	0.022
PTV D95 (> 60 Gy vs < 60 Gy)	0.31 (0.13–0.76)	0.0077
GTV V160_BED10_ (> 95% vs < 95%)	0.39 (0.18–0.87)	0.021
CTV V160_BED10_ (> 95% vs < 95%)	0.44 (0.20–0.97)	0.041
PTV V160_BED10_ (> 95% vs < 95%)	0.47 (0.21–1.03)	0.058
GTV D2% (> 65 Gy vs < 65 Gy)	0.33 (0.15–0.72)	0.0055
GTV D2%_BED10_ (> 165 Gy vs < 165 Gy)	0.32 (0.15–0.70)	0.0044

**Table 5:** Multivariable analysis: competing risk regression for LC, Cox regression for lung PFS and OS (distant PFS . Hazard ratio and *p*-value are reported only for the variables included in the multivariable analysis

*Endpoint*	Local control	Lung PFS		Overall survival		
*Variables*	Hazard ratio (95% CI)	*p* value	Hazard ratio (95% CI)	*p* value	Hazard ratio (95% CI)	*p* value
Primary histology (adeno vs other)	0.75 (0.28–2.02)	0.57	0.54 (0.28–1.07)	0.078	0.57 (0.22–1.50)	0.25
Primary tumor site (reference: Lung)						
GI vs Lung	0.55 (0.14–2.09)	0.38	0.90 (0.43–1.88)	0.77	0.29 (0.08–1.02)	0.05
Other vs Lung	1.00 (0.39–2.53)	0.99	0.61 (0.27–1.38)	0.24	0.37 (0.11–1.31)	0.12
Prior chemotherapy (yes vs no)	3.01 (0.76–11.92)	0.12	/	/	/	/
Age > 65 (yes vs no)	0.64 (0.24–1.67)	0.36	/	/	/	/
Performance status(1+ vs 0)	4.00 (0.86–18.64)	0.077	/	/	2.46 (0.56–10.74)	0.23
Gender (male vs female)	2.17 (0.86–5.44)	0.10	/	/	/	/
GTV volume (> 10 cc vs < 10 cc)	1.92 (0.78–4.75)	0.16	/	/	/	/
BED prescription (> 120 Gy vs < 120 Gy)	0.35 (0.14–0.87)	0.025	/	/	/	/

### Toxicity

The SBRT treatment was extremely well tolerated. Fiducial related events were low with 7% grade 1 and 2% grade 2 fiducial related pneumothorax without incidences of bleeding. The vast majority of patients developed no toxicity or grade 1 acute and late toxicity. Toxicity was not correlated with tumor location nor with the number of treated lesions. Only 1 patient developed acute grade 3 radiation pneumonitis (RP) and 2 patients grade 2. Regarding late toxicity, RP was slightly worse: two patients developed grade 3 and 1 patient with a centrally located lesion developed grade 4 RP with dyspnea and eventually died due to possible RT-induced pulmonary hemorrhage some 3 month after SBRT treatment. The treatment plan of the patient was analyzed and all treatment parameters and constraints were well below tolerance. The patient had various co-morbidities and was extensively pre-treated with various systemic treatments including bevacizumab.

## Discussion

The management of patients with overt distant metastases from solid tumors is usually considered palliative and are generally treated systemically depending on the primary disease. However, in case of OMLD, metastasis-directed therapy (MDT) has led to excellent long-term survival rates. Indeed, numerous (non-randomized) studies have shown the efficacy and benefit of surgical resections of lung metastases with reported 5-year OS rates up to 68% [1, 2]. Randomized trials such as the PulMiCC trial are currently ongoing to evaluate the effect of pulmonary metastasectomy on survival in advanced colorectal cancer [25]. However, in case of surgical or medical inoperability [26], these patients should not be denied MDT. SBRT is a well-documented non-invasive alternative to metastasectomy for a wide range of primary tumors and metastatic locations [8, 20, 27–29]. We present our report of 104 patients with 132 exclusively oligorecurrent lung metastases, all treated consistently with a Cyberknife® system.

Our 1, 2 and 3-year LC rates are in line with reported data on SBRT for lung metastases [30, 31]. In the literature, controversy remains on which characteristics (patient, tumor as well as planning) affect LC. For instance, there is debate on the impact of primary tumor etiology on LC [32–34]. Several studies report a worst LC for lung metastases from primary GI origin while Guckenberger et al. [35] did not observe an influence of different primary tumor histologies on LC. In our series, lung metastases from primary GI origin (34% of the lesions) showed a superior LC rate compared to primary lung (47% of the lesions) as well as the other mixed histologies. A possible explanation could be our rather limited number of treated lesions from primary GI etiology and limited variety of histologies of our cohort. The same controversy applies on the impact of metastatic tumor volume on LC [19, 31, 35, 36]. Our data shows a significant effect (in univariable analysis) of metastatic gross tumor volume on LC at the cut-off volume of 10 cc. Although other cut-off values have been proposed, our data support the hypothesis that metastatic volume significantly affects LC [19, 36]. In analogy to SBRT for primary NSCLC, a dose-response relationship has been shown for local tumor control [35]. However, due to a wide variety of treatment techniques, dose fractionation schemes and primary tumors, there remains uncertainty about the optimal dose to irradiate pulmonary metastases. We addressed this particular issue and suggest a biological effective dose (BED10) of 160Gy on the GTV (and CTV) as a critical threshold to significantly improve LC. This dose is at somewhat the higher end of the proposed threshold dose from the literature.

In our cohort, SBRT resulted in a 1, 2 and 3-year overall survival rates of 92.2, 80.9 and 72.0% respectively. Although to be taken with caution, these OS data compare favourably with other series on SBRT for lung metastases [37] and are at least comparable with those on metastasectomy [38]. A possible explanation for these results might be the high local control rates achieved by SBRT in our serie. In analogy to metastasectomy, patients with a complete resection had a significantly longer OS versus patients where only an incomplete resection was achieved [1]. Moreover, recent randomized trials in the setting of patients with oligometastatic cancers with a controlled primary [39] and patients with OM-NSCLC [40], (consolidative) local therapy was associated with a significant improvement of the OS. These data supports our observations and stresses the importance of local control in obtaining favourable OS in the ORLM setting. Moreover, our OS data tend to confirm previously published results which showed significantly higher OS rates in an ORLM setting when compared to OMLD patients in general [12, 41, 42]. Other relatively favourable clinical characteristics of our patients according to the Niibbe-Onishi-Chang classification [43] could have added to the favourable OS**:** 77% of our patients had only 1 metastasis in one site, i.e. lung, mostly from primary NSCLC (47%) and GI (34%). In analogy with improved LC, superior OS rates were observed from primary GI site in line with selected literature [44]. Furthermore, patients with a second de-novo metachronous-or repeat ORLM, a new SBRT session was the treatment of choice while in case of polymetastatic progression, patients received next generation systemic therapy and/or targeted agents, which could both add a survival benefit to our cohort. In our study population, out of field lung and distant metastasis were the most important form of treatment failure after SBRT. While a number of patients with repeat ORLM were eligible for subsequent SBRT treatment, the role of adjuvant systemic treatment in this ORLM setting is unclear. Further trials are warranted to evaluate the benefit of adjuvant therapy in this setting.

Our reported low toxicities are in line with previously published data after Cyberknife treatment for primary and metastatic disease [19] and comparable (including the dosimetrical parameters) with treatments performed on a classical linac-based SBRT platform [37, 45].

Limitations of the current investigation include its retrospective nature as well as the variations in treatment schedules, delivered dose, number and limited variations of the primary tumor sites of the treated lesions. Moreover, variability and quantity of previous local and systemic treatments might bias our outcome parameters due to the possible selection of radioresistant cells [46]. No control group was available to compare SBRT with other local treatments such as radiofrequency- and microwave ablation or surgery. Furthermore, only 62 lesions had a confirmed histology. Finally, due to referrals from different institutions, no standardized follow-up imaging protocol was used.

## Conclusions

In conclusion, SBRT as delivered in this study is a highly effective local therapy for the treatment of de-novo metachronous-and repeat oligorecurrent lung metastases and might be able to sterilize a limited number of lesions and achieve long term survival rates in patients with more favourable prognostic features.

## Data Availability

The datasets used and/or analysed during the current study are available from the corresponding author on reasonable request

## Abbreviations

SBRT: Stereotactic body radiotherapy
LC: Local control
Di-PFS: Distant progression free survival
OS: Overall survival
OMLD: Oligometastatic lung disease
ORLM: Oligorecurrent lung metastases
CT: Computed tomography
18F FDG PET: Fluorodeoxyglucose positron emission tomography
GTV: Gross tumor volume
CTV: Clinical target volume
PTV: Planning target volume
OAR: Organs at risk
CR: Complete response
PR: Partial response
SD: Stable disease
BED: Biological effective dose
CI: Confidence interval
Gy: Gray
MDT: Metastasis-directed therapy
GI: Gastro-intestinal
RP: Radiation pneumonitis
ILD: Interstitial lung disease
IPF: Idiopathic pulmonary fibrosis
